# Genetic susceptibility of cervical cancer

**DOI:** 10.1016/S1674-8301(11)60020-1

**Published:** 2011-05

**Authors:** Xiaojun Chen, Jie Jiang, Hongbing Shen, Zhibin Hu

**Affiliations:** a Department of Gynecology, Tumor Hospital of Nantong, Nantong, Jiangsu 226000, China; b Department of Epidemiology and Biostatistics, Nanjing Medical University, Nanjing, Jiangsu 210029, China;; c Section of Clinical Epidemiology, Jiangsu Key Lab of Cancer Biomarkers, Prevention and Treatment, Cancer Center, Nanjing Medical University, Nanjing, Jiangsu 210029, China.

**Keywords:** cervical cancer, genetic, human papillomavirus

## Abstract

Epidemiological and laboratory-based studies have identified infection with one of 15 high-risk human papillomavirus (HPV) types as a necessary but not sufficient cause of cervical cancer. The prevalence of genital HPV infections is high in young women, but most of the infections regress without interventions. Host genetic variations in genes involved in immune response pathways may be related to HPV clearance, and HPV E6/E7 oncoproteins interacting or downstream genes, both coding and non-coding, may contribute to the outcome of high risk HPV infection and cervical cancer. Of specific interest for this review has been the selection of genetic variants in genes involved in the above-referred pathways with a summary of their applications in association studies. Because the supportive and opposing data have been reported in different populations, well-designed international collaborative studies need to be conducted to define the consistency of the associations, paving the way to better define the patients at high risk of developing cervical cancer.

## INTRODUCTION

In spite of substantial declines in both incidence and mortality rates in the past 50 years, cervical cancer remains the second most common cancer among women worldwide, with estimated 493,000 new cases and 274,000 deaths in 2002[1]. Cervical screening has greatly reduced the incidence of invasive cervical cancer in many industrialized nations; however, the huge benefits have not been sufficiently available to countries in the developing world even though 80% of cervical cancer occurs in these countries[1].

Persistent cervical infection with one of 15 carcinogenic human papillomavirus (HPV) types causes invasive cervical cancer and also precursor abnormalities[2]. To date, two HPV vaccines, Gardasil and Cervarix, composed of noninfectious, recombinant HPV viral-like particles have been developed. The two vaccines potentially provide protection against the two most common types of carcinogenic HPV that cause approximately 70% of invasive cervical cancers worldwide: HPV 16 and 18. Preliminary analysis suggests that the reduction of the vaccine on HPV type 16/18-related cervical intraepithelial neoplasia (CIN) 2 and CIN3 or adenocarcinoma *in situ* is up to 39%[3]. However, no significant benefit was observed in women who were already infected with HPV[3]. Questions and concerns have also been raised about the duration of HPV vaccine-induced immunity, and whether other carcinogenic HPV types not contained in the vaccines will replace HPV types 16 and 18 in the ecological niche. Furthermore, the high cost of HPV vaccines available to date limits people living in the developing countries who would benefit most from being vaccinated or having an access to the vaccines[4].

HPV infection is necessary but not sufficient for the development of cervical cancer, because most individuals (70% to 90%) eliminate the virus 12 to 24 months after initial diagnosis without intervention[5],[6]. Evidence for familial aggregation in cervical cancer incidence has demonstrated that the risk associations reported were the strongest for full relatives, intermediate for half siblings, and the lowest for nonbiological relatives[7]. Therefore, molecular epidemiological studies are needed to identify host genetic factors and to better define the patients at an increased risk of developing cervical cancer as the target population that should receive cervical screening and HPV vaccines, which may improve the cost-effectiveness of cervical cancer prevention.

Remarkable progress has been made in this field, but overwhelming criticism is also received because of an initially overoptimistic view on the magnitude of the effects of inherited genetic variants. Given mounting evidence that long-term HPV infection of carcinogenic types is a prerequisite for cervical carcinogenesis, host genetic differences in immune-responsive genes and HPV E6/E7 oncoprotein-interacting and downstream genes may be mainly modifiers for cervical cancer susceptibility. Of specific interest in this review has been the selection of several well-characterized genetic variants involved in immune-responsive genes and HPV E6/E7 oncoprotein-interacting or downstream genes to give a summary of their applications in association studies.

## IMMUNE RESPONSE GENES

Available data indicate that cervical cancer develops through infection to preinvasive lesions to invasive cancer, a process that on average takes decades[8]. However, the molecular mechanisms involved in the development of a persistent HPV infection resulting in the development of cervical cancer are not well-established. Host innate immunity and adaptive immune response all are responsible for the regression, persistence, or progression of HPV infection. For example, a polarization of the immune response for T-helper 2 (Th2) was observed in women with HPV infections that evolve into high-grade lesions[9], whereas squamous intraepithelial lesions and *in situ* carcinomas evading immunological control seem to be influenced by the cellular antigen presentation system and the HLA receptors[5]. The transcriptional blockage of the HPV DNA also facilitates the malignant transformation caused by HPV persistent infection, and in this case, the tumor necrosis factor-α (TNF-α)-mediated cascade plays an important role[5].

### Interleukin-1B gene (*IL*-*1B*)

IL-1β, encoded by the *IL*-*1B* gene, is a potent pro-inflammatory cytokine and an important part of the innate immune system. Studies have shown that IL-1β plays an important role in cervical carcinogenesis[10],[11]. Our previous study revealed that the mean plasma IL-1β levels in cervical cancer cases were significantly higher than those in controls (*P* = 0.0002), and plasma IL-1β levels above the 75% quartiles in controls were associated with a 1.74-fold significant increase in the risk of cervical cancer[12]. Inter-individual variation in IL-1β levels appears to be influenced in part by functional polymorphisms in the transcription regulatory regions of *IL*-*1B*[12],[13]. Several case-control studies have been conducted to determine the roles of *IL*-*1B* promoter single nucleotide polymorphisms (SNPs) in the development of cervical cancer, especially for C-511T (rs16944)[12],[14],[15]. As shown in ***Table 1***, the *IL-1B*-511T and -31C alleles have been found to confer an increased risk for the development of cervical cancer, especially among subjects having higher levels of IL-1β[12].

**Table 1:** Published studies on immune response genes and risk of cervical cancer

Gene	SNP	Case/Control	Genotype	OR (95% CI)	Ref
*IL-1B*	C-511T (rsl6944)	404/404	CC	Ref	Qian N 2010
			CT	1.53 (1.09-2.15)	
			TT	1.47 (0.97-2.24)	
			CT/TT	1.52 (1.10-2.09)	
		182/364	CC	Ref	Kang S 2007
			CT	2.83 (1.52-5.28)	
			TT	1.68 (0.85-3.32)	
			CT/TT	2.42 (1.31-4.46)	
		150/162	CC	Ref	Singh H 2008
			CT	1.37 (0.59-3.20)	
			TT	2.77 (1.21-6.41)	
			CT/TT	2.00 (0.96-4.16)	
	T-31C (rs1143627)	404/404	TT	Ref	Qian N 2010
			TC	1.65 (1.17-2.31)	
			CC	1.50 (0.99-2.26)	
			TC/CC	1.60 (1.16-2.21)	
*TNFA*	G-308A (rs1800629)	1263/794	GG	Ref	Ivansson EL 2010
			GA	1.20 (0.97-1.47)*	
			AA	0.78 (0.46-1.32)*	
			GA/AA	1.14 (0.94-1.39)*	
		244/228	GG	Ref	Govan VA 2006
			GA	1.33 (0.86-2.06)*	
			AA	0.79 (0.30-2.05)*	
			GA/AA	1.24 (0.82-1.86)*	
		195/244	GG	Ref	Duarte I 2005
			GA	1.81 (1.10-2.97)	
			AA	2.54 (0.65-10.5)	
			GA/AA	1.88 (1.20-2.94)	
		103/101	GG	Ref	Stanczuk GA 2003
			GA	1.70 (0.87-3.33)*	
			AA	0.55 (0.05-6.16)*	
			GA/AA	1.59 (0.83-3.04)*	
		150/162	GG	Ref	Singh H 2009
			GA	1.86 (0.84-4.13)*	
			AA	3.31 (1.03-10.7)*	
			GA/AA	2.24 (1.14-4.40)	
		127/108	GG	Ref	Calhoun ES 2002
			GA	0.72 (0.40-1.32)	
			AA	1.81 (0.53-6.10)	
			GA/AA	0.85 (0.48-1.49)*	
		115/113 Hispanic	GG	Ref (random control)	Deshpande A 2005
			GA	0.79 (0.42-1.49)*	
			AA	2.25 (0.56-9.00)*	
			GA/AA	0.93 (0.52-1.69)*	
		143/119 no-Hispanic white	GG	Ref (random control)	Deshpande A 2005
			GA	0.90 (0.51-1.60)*	
			AA	2.53 (0.66-9.64)*	
			GA/AA	1.05 (0.61-1.80)*	
		115/104 Hispanic	GG	Ref (HPV16 + control)	Deshpande A 2005
			GA	0.92 (0.47-1.79)*	
			AA	2.14 (0.54-8.58)*	
			GA/AA	1.10 (0.60-2.05)*	
		143/75 no-Hispanic white	GG	Ref (HPV16 + control)	Deshpande A 2005
			GA	0.73 (0.38-1.40)*	
			AA	1.03 (0.33-3.23)*	
			GA/AA	0.76(0.43-1.38)*	
*IL-12B*	3′UTR A>C rs3212227	404/404	AA	Ref	Chen X 2009
			AC	1.29 (0.94-1.77)	
			CC	1.33 (0.88-2.00)	
			AC/CC	1.30 (0.97-1.75)	
		150/179	AA	Ref	Han SS 2008
			AC	1.61 (0.94-2.73)	
			CC	1.29 (0.68-2.46)	
			AC/CC	1.51 (0.91-2.51)*	
		200/200	AA	Ref	Kordi Tamandani DM 2009
			AC	1.80 (1.17-2.78)	
			CC	--	
			AC/CC	1.71 (1.11-2.63)	
*IFNG*	A+874T (rs2430561)	1290/802	AA	Ref	Ivansson EL 2010
			AT	1.36 (1.11-1.66)*	
			TT	1.41 (1.10-1.81)*	
			AT/IT	1.37 (1.05-1.23)*	
		200/230	AA	Ref	Gangwar R 2009
			AT	0.64 (0.42-0.98)*	
			TT	0.42 (0.23-0.75)*	
			AT/TT	0.58 (0.39-0.86)*	
		200/200	AA	Ref	Kordi Tamandani MK 2008
			AT	3.27 (2.10-5.09)	
			TT	1.88 (0.98-3.62)	
			AT+TT	2.92 (1.92-4.45)	
		261/405	AA	Ref	Govan VA 2003
			AT	1.53 (1.09-2.14)*	
			TT	1.03 (0.56-1.87)*	
			AT/TT	1.41 (1.03-1.94)*	
*IL-10*	A-1082G (rs1800896)	667/606	AA	Ref	Zoodsma M 2005
			AG	0.90 (0.68-1.19)*	
			GG	0.93 (0.68-1.28)*	
			AG/GG	0.91 (0.70-1.19) *	
		197/182	AA	Ref	Govan VA 2003
			AG	1.06 (0.68-1.66)*	
			GG	0.61 (0.35-1.08)*	
			AG/GG	0.89 (0.59-1.33)*	
		77/69	AA	Ref	Stanczuk GA 2001
			AG	3.63 (1.65-8.01)*	
			GG	--	
			AG/GG	3.75 (1.71-8.24)*	
*CTLA-4*	G+49A (rs231775)	696/709	GG	Ref	Hu L 2010
			GA	1.03 (0.82-1.29)	
			AA	1.66 (1.13-2.44)	
		139/375	GG	Ref	Su TH 2007
			GA	1.19 (0.78-1.80)*	
			AA	1.20 (0.64-2.27)*	
		141/217	GG	Ref	Pawlak E 2010
			GA	1.13 (0.64-2.01)*	
			AA	0.98 (0.53-1.82)*	

* Calculated with frequency from the original paper without adjustment. Ref: reference; IL: Interleukin; *TNFA*: tumor necrosis factor-α gene; *IFNA*: interferou-γ gene; *CTLA-4*: cytotoxic T-lymphocyte antigen-4 gene.

### Tumor necrosis factor A gene (*TNFA*)

TNF-α, encoded by the *TNFA* gene, is another potent pro-inflammatory cytokine that has been implicated in the control of HPV infection. HPV-harboring cervical keratinocytes constitutively produce active TNF-α[16], whereas cervical cancer cell lines or cervicovaginal washing fluid from patients with cervical neoplasia all have high levels of TNF-α[11],[17]. Control of HPV infection by TNF-α occurs by induction of apoptosis in HPV-infected cells, arresting the growth of HPV infected keratinocytes, and down-regulating HPV transcription[18]–[20]. As for *TNFA* polymorphisms, the majority of published studies have been focused on G-308A (rs1800629) in the promoter region, because the -308A allele is thought to increase the transcriptional activity of the *TNFA* gene[21]. Studies on *TNFA* G-308A and risk of cervical cancer are shown in ***Table 1***, in which some of the reports, collectively, supported that the variant GA/AA genotypes were associated with an increased cervical cancer risk[22]–[26], although others did not[27],[28].

### Human leukocyte antigen genes (*HLA*)

HLA is essential for the presentation of viral antigens. The activity of HLA molecules seems to be one of the determining factors in the induction of an adaptive immune response[29]. Polymorphisms within *HLA* have been hypothesized to be involved in the pathogenesis of cervical neoplasia via their role in the immunological control of HPV infection. Case-control studies have shown associations between specific *HLA* alleles and cervical cancer risk[30],[31]. In a meta-analysis, Yang *et al*. reported that seven alleles (HLA DRB1*0403, *0405, *0407, *0701, *1501, *1502 and *1503) were positively associated with, while four other alleles (HLA DRB1*0901, *1301, *1302, and *1602) were negatively associated with cervical squamous cell carcinoma[32]. However, in a later published study, the HLA DRB1*1501-DQB1*0602 haplotype was reported to be protective against the high-grade CIN, especially in individuals infected with oncogenic HPV[33]. In addition, a positive association between HLA DQB1*0302 and cervical disease has also been demonstrated in several populations, including the cohort in Costa Rica[31].

### Interleukin-12 A and B gene (*IL12A* and ***IL12B***)

IL-12 is a heterodimeric pro-inflammatory cytokine formed by a 35,000 Dalton light chain (known as p35 encoded by *IL12A*) and a 40,000 Dalton heavy chain (known as p40 encoded by *IL12B*), which induces the production of interferon-γ (IFN-γ), favors the differentiation of Th1 cells and forms a link between innate and adaptive immunity[34]. Besides its antiviral activity, IL-12 is important for host resistance to carcinogenesis. The anticancer activity of IL-12 has been extensively reported in mouse models, where it has been shown to inhibit tumorigenesis and induce regression of established tumors[35]–[37]. We genotyped *IL*-*12A* rs568408 [3′ untranslated region (UTR) G>A] and rs2243115 (5′UTR T>G) and *IL*-*12B* rs3212227 (3′UTR A>C) in a hospital-based study of 404 cervical cancer cases and 404 cancer-free controls. The *IL*-*12A* rs568408 GA/AA and *IL*-*12B* rs3212227 AC/CC variant genotypes were associated with increased risks of cervical cancer [odds ratio (OR) = 1.43, 95% confidence interval (CI) = 1.06-1.93; and 1.30, 95% CI = 0.97-1.75, respectively], compared with their corresponding wild-type homozygotes[38]. As shown in ***Table 1***, the risk associated with *IL12B* rs3212227 is also supported by other published studies[39],[40].

### Interferous-γ gene (*IFNG*)

IFN-γ, encoded by the *IFNG* gene at 2q33, plays a pivotal role in defense against viruses and intracellular pathogens and in the induction of immune mediated inflammatory responses[41]. Pravica *et al*. reported that an A to T change, located at the +874 position from the translation start site in the first intron of *IFNG*, which coincides with a putative NF-κB binding site that may play a fundamental role in the induction of constitutively high IFN-γ production[42]. The variant T allele associated with higher IFN-γ levels was mostly reported to be associated with increased cervical cancer risk[22],[43],[44] with the exception of Gangwar et al.[45].

### Interleukin-10 gene (*IL*-*10*)

Studies have documented a profile of increased Th2 and decreased Th1 cytokine in premalignant cervical legions[46],[47]. IL-10 stimulates functions of innate and Th2-related immunity but suppresses Th1-related immune responses[48],[49]. In cervical cancer and CIN, the risk and the progression of cervical cancer have been associated with increased IL-10 serum levels[50],[51]. Three polymorphisms within the IL-10 promoter, at positions -1082, -819, and -592 have been identified[52]. The -1082 SNP has been found important in determining cytokine production with the variant GG associated with high IL-10 levels[52]. Although a significant association between the polymorphism and risk of cervical cancer has been reported in a small study[53], it was not confirmed in other studies[44],[54].

### Cytotoxic T-lymphocyte antigen-4 gene (*CTLA*-*4*)

Cytotoxic T-lymphocyte antigen-4 (*CTLA4*), located at 2q33, encodes a receptor expressed by activated T lymphocytes. SNPs in *CTLA4* have been reported to be associated with susceptibility to both autoimmune disease and cancer[55],[56]. Among these SNPs, the *CTLA4* G49A (rs231775) variant causes 17Thr-to-Ala substitution in the leading peptide of the CTLA4 receptor[57]. Recently, we and our collaborators reported that the 17Thr-to-Ala change in CTLA4 greatly enhanced the ability of the receptor to interact with its ligand B7.1, and recombinant CTLA4-17Ala had a significantly stronger ability to inhibit T-cell proliferation and activation compared with its counterpart CTLA4-17Thr[56]. In molecular epidemiological studies, *CTLA4* G49A was found to be significantly associated with risk of multiple types of cancer[55],[56]. However, several studies have investigated the relationship between the *CTLA4* G49A variation and cervical cancer risk but have not found any significant associations[58],[59]. Recently, we reported our results of cervical cancer and found that the genotype AA of *CTLA4* G49A was associated with a 1.66-fold (95% *CI*=1.13-2.44) increased cervical cancer risk[60].

### HPV E6/E7 oncoproteins interacting or downstream genes

HPV is a double strand DNA virus that contains around 8,000 base pairs. Among the different components of the HPV genome, the most commonly expressed viral proteins closely associated with cervical cancer development are the E6 and E7 oncoproteins[5], which bind to and degrade the host tumor suppressor proteins, p53 and Rb, respectively, and make the viral proteins override the functions of cell cycle checkpoints and cause cellular transformation[61].

#### p53

*p53* guards the integrity of human genome by regulating cell cycle arrest, DNA repair and apoptosis. It is well characterized that the HPV oncoprotein E6 can degrade p53 through the ubiquitin pathway, resulting in chromosomal instability and cellular malignant transformation[62]. One known common SNP is codon 72 of p53, with two alleles encoding either arginine or proline. Since Storey *et al*.[63] reported that individuals homozygous for p53Arg were seven times more susceptible to HPV-associated cervical carcinogenesis than heterozygotes, many groups have investigated the effect of p53 codon 72 polymorphism on cervical cancer risk. A recently published meta-analysis including 49 studies showed that pooled estimates for invasive cervical cancer were 1.22 (95%*CI* = 1.08-1.39) for arginine homozygotes, compared with heterozygotes, and 1.13 (95%*CI* = 0.94-1.35) for arginine homozygotes versus proline homozygotes. However, further subgroup analyses showed that significant excess risks were only observed in studies where controls were not in Hardy-Weinberg equilibrium, in non-epidemiological studies, and in studies where the p53 genotype was determined from tumor tissues[64]. Consistently, we did not find a significant association between p53 codon 72 polymorphism and cervical susceptibility[65].

### Breast cancer susceptibility gene 1 (*BRCA1*) and BRCA1-associated ring domain protein 1 (*BARD1*) gene

It was reported that both the E6 and E7 oncoproteins interacted directly with and functionally antagonized BRCA1[66] that binds to p53 and acts as a co-activator of the p53-mediated transcription[67],[68]. BARD1 has been described as a nuclear protein that interacts with BRCA1 and forms a stable BRCA1-BARD1 complex in the nucleus, which was also identified to interact with HPV E6 to lead E6 inactivation and to stabilize p53 in cervical carcinogenesis[69]. In a case-control study of cervical cancer, we genotyped *BRCA1* Pro871Leu (rs799917), *BARD1* Pro24Ser (rs1048108) and Arg378Ser (rs2229571) and found that the *BRCA1* variant rs799917 TT genotype was associated with a significantly decreased risk of cervical cancer in a recessive genetic model (*OR*=0.62, 95%*CI*=0.40-0.95), but this association was not observed for the two *BARD1* SNPs[65].

### Primary microRNA-218 (*pri*-*miR*-*218*) and laminin-5 β3 (*LAMB3*)

Except for affecting protein coding genes, the expression of the E6 oncoprotein of the high-risk HPV16 could also reduce microRNA-218 (miR-218) expression[70]. Conversely, RNA interference of E6/E7 oncogenes in an HPV 16 positive cell line could increase miR-218 expression[70]. Laminin-5 is required in (RAS) and NF-κB blockade-induced tumorigenesis of human squamous cell carcinoma[71] and has been considered a marker of invasiveness in cervical lesions[72]. LAMB3 has been verified as a transcriptional target of miR-218[70], and the expression of LAMB3 is increased in the presence of the HPV16 E6 oncoprotein, which is mediated through miR-218[70]. A recent study indicated that secreted laminin-5 could be used by HPV virus as a transient receptor to aid the virus in the infection of basal cells that express α6β4-integrin[73]. Thus, downregulation of *miR*-*218* by E6 and the consequent over expression of LAMB3 may promote viral infection of the surrounding tissue and eventually contribute to cervical carcinogenesis. In a recent case-control study, we identified one SNP rs11134527 located in the pri-miR-218 sequence and one SNP rs2566 in the 3′ UTR of LAMB3 and found that the pri-miR-218 rs11134527 variant homozygote GG was associated with a decreased risk of cervical cancer (*OR*=0.72, 95%*CI*=0.52-0.99), while the LAMB3 rs2566 variant CT/TT genotypes were associated with a significantly increased risk of cervical cancer (*OR*=1.57, 95%*CI*=1.25-1.96), although the functional relevance of these two SNPs have not well characterized[74].

### Other genes related to HPV persistence and progression

Globally, overall HPV infection has accounted for the persistence (*P* = 0.01) and for CIN3 or worse (*P* = 0.07). Excluding HPV16, the *P* values would be 0.04 and 0.37, respectively. For HPV16, non-European viral variants were significantly more likely than European variants to cause persistence (*OR*=2.6; *P* = 0.01) and CIN3 or worse (*OR*=2.4; *P* = 0.004)[75]. Although it is known that host genetic factors may be responsible for a subset of individuals infected with these oncogenic viruses to have persistent infection and to develop CIN3 or cervical cancer, whereas the vast majority of infected individuals naturally clear their infections, the underlying molecular mechanisms are poorly understood.

A population-based cohort study of 10,049 women in Guanacaste, Costa Rica, performed analyses on several candidate SNPs in selected immune response and DNA repair genes to evaluate variants involved in HPV persistence and progression[76],[77]. Wang *et al*. found that some SNPs of DNA repair genes including *IRF3* Ser427Thr (rs7251), *EXO1* Thr439Met (rs4149963), *CYBA* 3′UTR (rs7195830), and *FANCA* Gly501Ser (rs2239359), were demonstrated to increase risk for CIN3 or cervical cancer, whereas immune-responsive gene *TLR2* Ser450Ser (rs3804100) and DNA repair gene *XRCC1* Gln399Arg (rs25487) polymorphisms were demonstrated to decrease risk. Furthermore, *IRF3* S427T and *XRCC1* Gln399Arg polymorphisms were significantly associated with HPV persistence, whereas *TLR2* Ser450Ser, *EXO1* Thr439Met, *CYBA* 3′UTR, and *FANCA* polymorphisms were associated with the risk of progression to CIN3 or worse[76].

In a subsequent study by the same group, genes/regions statistically significantly associated with CIN3 or cervical cancer included viral infection and cell entry genes *OAS3*, *SULF1*, and *IFNG*; the DNA repair genes *DU, DMC*, and *GTF2H4*; the *EVER1* and *EVER2* genes. *OAS3, SULF1, DUT*, and *GTF2H4* SNPs were associated with HPV persistence, whereas *IFNG* and *EVER1/EVER2* SNPs were associated with progression to cervical cancer[77]. However, further studies are needed to validate and expand these results.

## CONCLUSION

Although previous studies of potentially functional polymorphisms in candidate genes and cervical cancer susceptibility showed lack of consistence, they have advanced our knowledge of the genetic basis of the etiology of this cancer. For the association of low-penetrance genetic variants and cancer risk, heterogeneity is a great challenge, possibly coming from the variations in study design, ethnic diversity of target populations, lack of adjustment for known risk factors, small or modest sample sizes, and issues related to multiple significance testing. Therefore, future studies should call for a more careful study design, study execution, and data analysis. Specific for cervical cancer, a better understanding of the natural history of oncologic HPV infection and factors related to HPV persistence and progression will facilitate the identification of susceptible subjects.

With high-throughput genotyping methods available, genome-wide association (GWA) approaches are popular in recent years, which may provide us the opportunity to give a comprehensive genetic view of the disease. However, up to now, GWA study was reported for neither cervical cancer nor HPV persistence. Most published studies have not presented sufficient information on environmental exposure other than HPV infection, which may also contribute to the heterogeneity between studies and populations. An important aspect of epidemiological studies is the ability to form improved multicenter research consortia, which may be beneficial for not only increasing study sample sizes, but also for providing finance, ensuring data quality and generalizing the findings and conclusions.

The past years have seen that the GWA approach is well-suited for the identification of common SNPs with modest or small effects on the well-defined phenotypes[78]. However, we are far from being able to explain a relative large proportion of observed familial clustering for most multifactorial traits. Although the relative impact of common and rare variants on common diseases remains an unanswered empirical question, we think that rare variants could also be the primary drivers of common diseases. Then, whole-genome and whole-exome sequencing based GWA approach, for the discovery of rare causal variants, may come forth in the near future and family-based or extreme-trait designs may help add to the power[79].
